# Disposable screen printed sensor for the electrochemical detection of methamphetamine in undiluted saliva

**DOI:** 10.1186/s13065-016-0147-2

**Published:** 2016-02-01

**Authors:** Carrie-Ann Bartlett, Sarah Taylor, Carlos Fernandez, Ceri Wanklyn, Daniel Burton, Emma Enston, Aleksandra Raniczkowska, Murdo Black, Lindy Murphy

**Affiliations:** Oxtox Limited, Warren House, 5 Mowbray Street, Stockport, SK1 3EJ UK

**Keywords:** Square wave voltammetry, SWV, Galvanostatic oxidation, Screen printed electrode, Mediator, Methamphetamine, Saliva, Detection

## Abstract

**Background:**

Methamphetamine has an adverse effect on the ability to drive safely. Police need to quickly screen potentially impaired drivers therefore a rapid disposable test for methamphetamine is highly desirable. This is the first proof-of-concept report of a disposable electrochemical test for methamphetamine in undiluted saliva.

**Results:**

A screen printed carbon electrode is used for the *N*,*N*′-(1,4-phenylene)-dibenzenesulfonamide mediated detection of methamphetamine in saliva buffer and saliva. The oxidized mediator reacts with methamphetamine to give an electrochemically active adduct which can undergo electrochemical reduction. Galvanostatic oxidation in combination with a double square wave reduction technique resulted in detection of methamphetamine in undiluted saliva with a response time of 55 s and lower detection limit of 400 ng/mL.

**Conclusions:**

Using a double square wave voltammetry technique, rapid detection of methamphetamine in undiluted saliva can be achieved, however there is significant donor variation in response and the detection limit is significantly higher than desired. Further optimization of the assay and sensor format is required to improve the detection limit and reduce donor effects.

**Electronic supplementary material:**

The online version of this article (doi:10.1186/s13065-016-0147-2) contains supplementary material, which is available to authorized users.

## Background

Two thirds of US trauma centre admissions are due to motor vehicle accidents with almost 60 % of such patients testing positive for drugs or alcohol [1]. Cannabis, cocaine and methamphetamine are the drugs most frequently detected in drivers randomly stopped for roadside drug screening [2–5]. In Norway prior to the year 2000 there was almost no methamphetamine on the Norwegian market. There was a steady increase in methamphetamine usage till 2010 where it appeared to have stabilized. The data for this study was confirmed by testing venous blood of convicted motorists, customs seizures and wastewater analysis [6]. A US survey, using a questionnaire which annually monitored adolescent drug use, showed a gradual decline in methamphetamine use from 3.7 % in 1981 (peak year) to 1.2 % in 2008 [7]. A recent study showed conflicting trends when comparing the questionnaire survey approach and wastewater analysis. Over the period 2010–2013 the population survey showed a slight decline in methamphetamine use while wastewater analysis showed a doubling of methamphetamine usage [8].

Methamphetamine remains a significant public health concern with known neurotoxic and neurocognitive effects to the user [9]. It is frequently abused as a recreational drug due to its stimulant and euphoric effects. The physiological and psychological side effects of methamphetamine include confusion, paranoia, depression, nausea and blurred vision. Driving a vehicle while under the influence of methamphetamine is thus clearly undesirable.

Roadside screening for methamphetamine in oral fluid has a number of requirements: it needs to be fast, ideally 15–30 s, i.e. ideally the same speed as a breath alcohol test; it must be very sensitive, ideally <10 ng/mL (25 ng/mL was used as the cut-off concentration in the European DRUID [Driving under the influence of Drugs, Alcohol, and Medicines] project [5]); and it should be non-invasive, difficult to tamper with and be portable. The currently available drug screening products require a minimum of 5–10 min for a test [10]. Test time and cost are restricting the roadside drug screening market to <10 % the volume of the alcohol screening market.

Oral fluid which contains saliva and other liquid substances present in the oral cavity are of great interest for roadside drug screening. Although this fluid is easy to collect there is considerable inter-sample variability in the fluid matrix that generates issues when developing a testing methodology [11]. Dilution of the sample can reduce the donor variability, however this dilutes the drug of interest and therefore requires the device to have greater sensitivity. The current devices on the market are primarily lateral flow immunodiagnostic tests, where the presence or absence of a coloured bar can be read either visually or in a meter in response to the drug of interest; these were used in the DRUID project. The response times are typically several minutes. The clinical sensitivity of these devices in saliva can be relatively poor at 16–75 % although clinical specificity can be close to 100 % [12].

There are only a few reports of the electrochemical sensing of amphetamines, and there are no reports of the determination of amphetamines in undiluted saliva using disposable electrochemical sensors. Electrochemical sensing of methamphetamine by direct oxidation has been reported at a pretreated pencil graphite electrode [LOD 50 nM (7.5 ng/mL) in aqueous solution, response time >10 min] [13], at a self-assembled boron-doped diamond electrode [LOD 50 nM (7.5 ng/mL) in aqueous solution, response time not given] [14], and in alkaline solution using a gold nanoparticle-multiwalled carbon nanotube modified screen printed electrode [LOD 0.3 nM (0.05 ng/mL), response time not given] [15]. The indirect electrochemical detection of amphetamine in saliva has been reported using 1,2-naphthoquinone-4-sulfonate at an edge plane pyrolytic graphite electrode [LOD 41 μM (6.2 μg/mL) in aqueous solution, response time not given] [16].

This paper reports a mediated screen printed carbon electrode for the detection of methamphetamine in undiluted saliva using substituted *N*,*N*′-(1,4-phenylene)-dibenzenesulfonamide mediator. Screen printed electrodes are well established as cheap and disposable single use sensors which can be manufactured with high reproducibility [17].

The sensor is optimized for speed of response and for response in undiluted saliva.

## Results and discussion

### Initial mediator screen

**Fig. 1:**
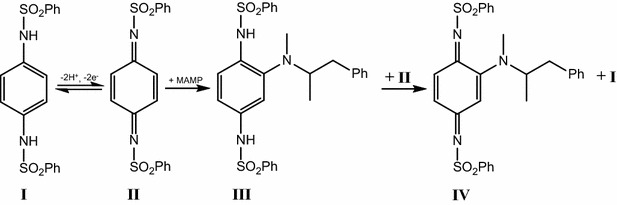
Reaction of *N*,*N*′-(1,4-phenylene)-dibenzenesulfonamide with methamphetamine (MAMP)

The mechanism of reaction between oxidized *N*,*N*′-(1,4-phenylene)-dibenzenesulfonamide and primary and secondary amines has been described by Adams and Schowalter [18]. The mechanism is shown schematically in Fig. 1. The oxidized form of the mediator (II) reacts with secondary amines such as methamphetamine (MAMP) by 1,4-addition resulting in the reduced form of the MAMP-mediator adduct (III). Electron exchange between (III) and a further molecule of (II) results in the oxidized form of the adduct (IV) which can undergo reduction at the electrode at the appropriate reduction potential i.e. it can give rise to a new reduction peak in addition to the reduction peak for unreacted oxidized mediator, (II).

With primary amines such as amphetamine (AMP), 1,2-addition can take place, resulting in elimination of the two benenesulfonamide groups from the mediator and formation of an AMP-mediator adduct. This adduct can also undergo oxidation via II and subsequently undergo electrochemical reduction.

**Fig. 2:**
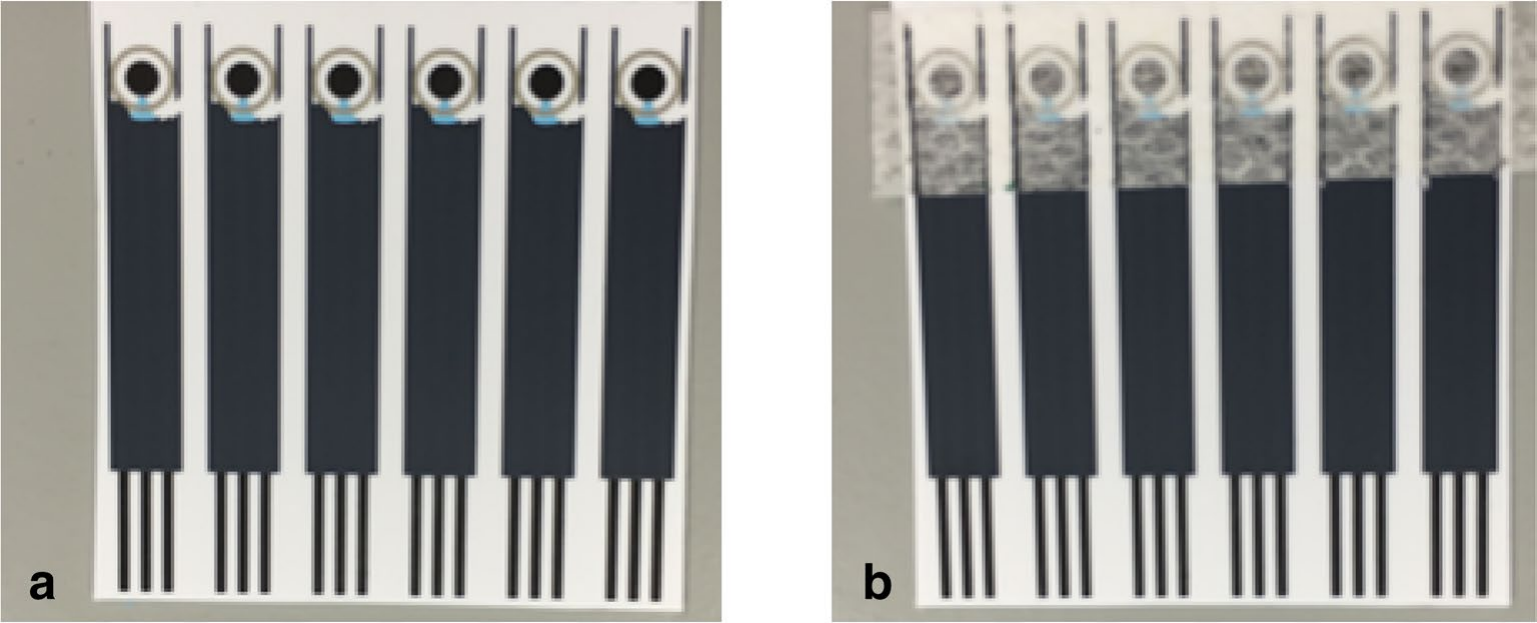
Screen printed electrodes **a** without and **b** with overlayer. The sensor comprises a circular carbon working electrode (2 mm diameter) and outer Ag/AgCl counter/reference electrode

An initial mediator screen was performed with several substituted *N*,*N*′-(1,4-phenylene)-dibenzenesulfonamide compounds, described in Additional file 1. The mediators were screened for electrochemical response using differential pulse voltammetry (DPV) and reaction with MAMP. The sensors used were of the format shown in Fig. 2a, consisting of a two electrode system of carbon working electrode and Ag/AgCl combined counter/reference electrode. The preferred mediator was OX1006 (*N*,*N*′-(2-nitro-1,4-phenylene)dibenzenesulfonamide) on the basis of giving a large, clearly defined peak response to MAMP without adsorption of the parent mediator to the electrode. At pH 10.8, the mediator is fully deprotonated [pKas 6.05 and 8.00 (25 °C)] and soluble at 1 mg/mL, and this pH was used for the development of the sensor.

**Fig. 3:**
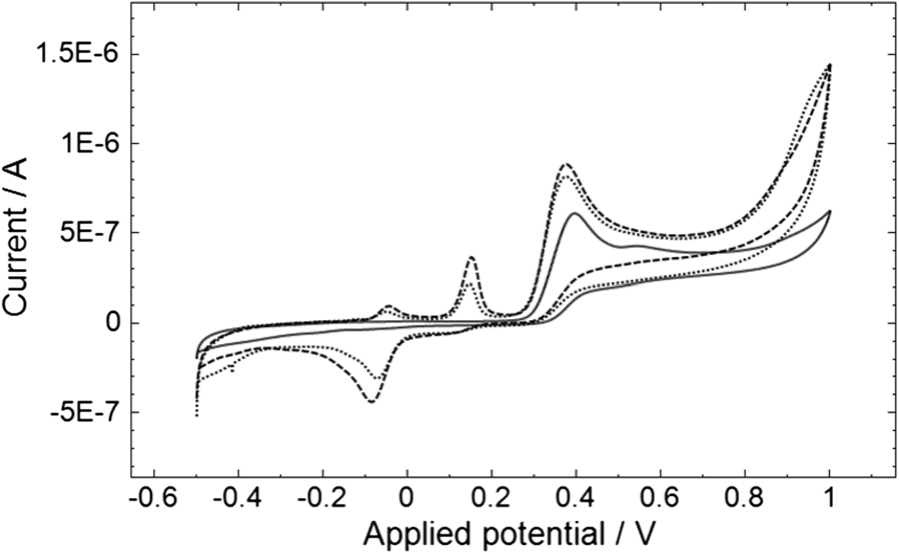
Cyclic voltammetry of OX1006 in the absence and presence of MAMP. The MAMP concentration was 0, 25 or 50 μg/mL MAMP (*solid line*, *dotted line* and *dashed lines*) in 0.1 M sodium carbonate buffer (pH 10.4), 0.2 M NaCl. 15 μL of solution was pipetted onto the sensor. The scan rate was 50 mV/s

The cyclic voltammetry of OX1006 with MAMP is shown in Fig. 3. In the absence of MAMP, there is a single oxidation peak at +0.38 V and negligible reduction peak. In the presence of MAMP, two new peaks are present at +0.15 V and −0.046 V, and a new reduction peak is present at −0.088 V. In addition, the parent mediator peak height at +0.38 V is increased by 29 and 47 % in the presence of 25 and 50 μg/mL MAMP. The increase in the parent peak height and the new peaks are due to the oxidation/reduction of the mediator-MAMP adduct.

### Optimization of electrochemical procedure with dried reagent

It was desired that the mediator and buffer solution be dried down in some way on the sensor. Deposition of mediator solution directly onto the sensor requires tight control of the volume and position of the dispensed reagent. Therefore an alternative technique was used comprising a porous overlayer onto which mediator was dried and which is then secured over the sensor. On application of sample, the mediator dissolves and diffuses to the working electrode where it can be oxidized, react with MAMP and produce a reduction response to MAMP. Sensors with overlayer applied are shown in Fig. 2b.

Galvanostatic oxidation of OX1006 was investigated in combination with the overlayer. The advantage of galvanostatic oxidation compared to potentiostatic oxidation is that the amount of oxidized mediator should be relatively independent of the concentration of mediator which has dissolved off the overlayer and reached the electrode surface, provided there is sufficient mediator. A potential disadvantage of galvanostatic oxidation is that if there is insufficient mediator, other species present will be oxidized, resulting in a large increase in working electrode potential.

There are very few reported examples of galvanostatic oxidation to generate reactant, and these examples are for electrochemical titrations using separate generator-collector electrodes [19, 20]. For example, Tomcik et al. [21] have reported the galvanostatic generation of hypobromite at an interdigitated microelectrode array, for end-point titration of the drugs Antabus and Celaskon. In our application, the working electrode is used to both generate the reactant (oxidized mediator) and detect the mediator-MAMP adduct.

**Fig. 4:**
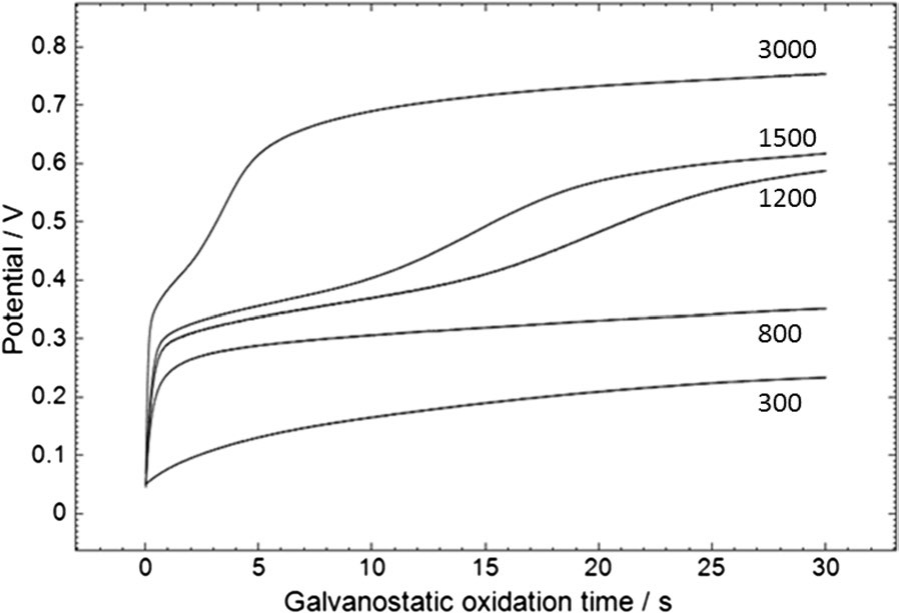
Varying the current during the galvanostatic oxidation step. The overlayer was treated with 0.12 mg/mL of OX1006 in 0.4 M sodium carbonate buffer (pH 10.8), containing 0.23 M NaCl and 0.1 % TX-100. The procedure consisted of a 10 s wait time after application of 7 μL of saliva, followed by galvanostatic oxidation at 300, 800, 1200, 1500 or 3000 nA for 30 s

The shift in working electrode potential during galvanostatic oxidation is shown in Fig. 4, for sensors with mediator in the overlayer and using a saliva sample. A 10 s wait time during which the sensor was at open circuit potential was employed at the start of the test sequence to ensure the mediator had dissolved off the overlayer. With higher galvanostatic currents there is a larger shift in potential starting at +0.4 V, with the shift seen at an earlier time for higher current, indicating the mediator has been depleted more quickly with higher galvanostatic current setting. A galvanostatic current of 800 nA was selected.

**Fig. 5:**
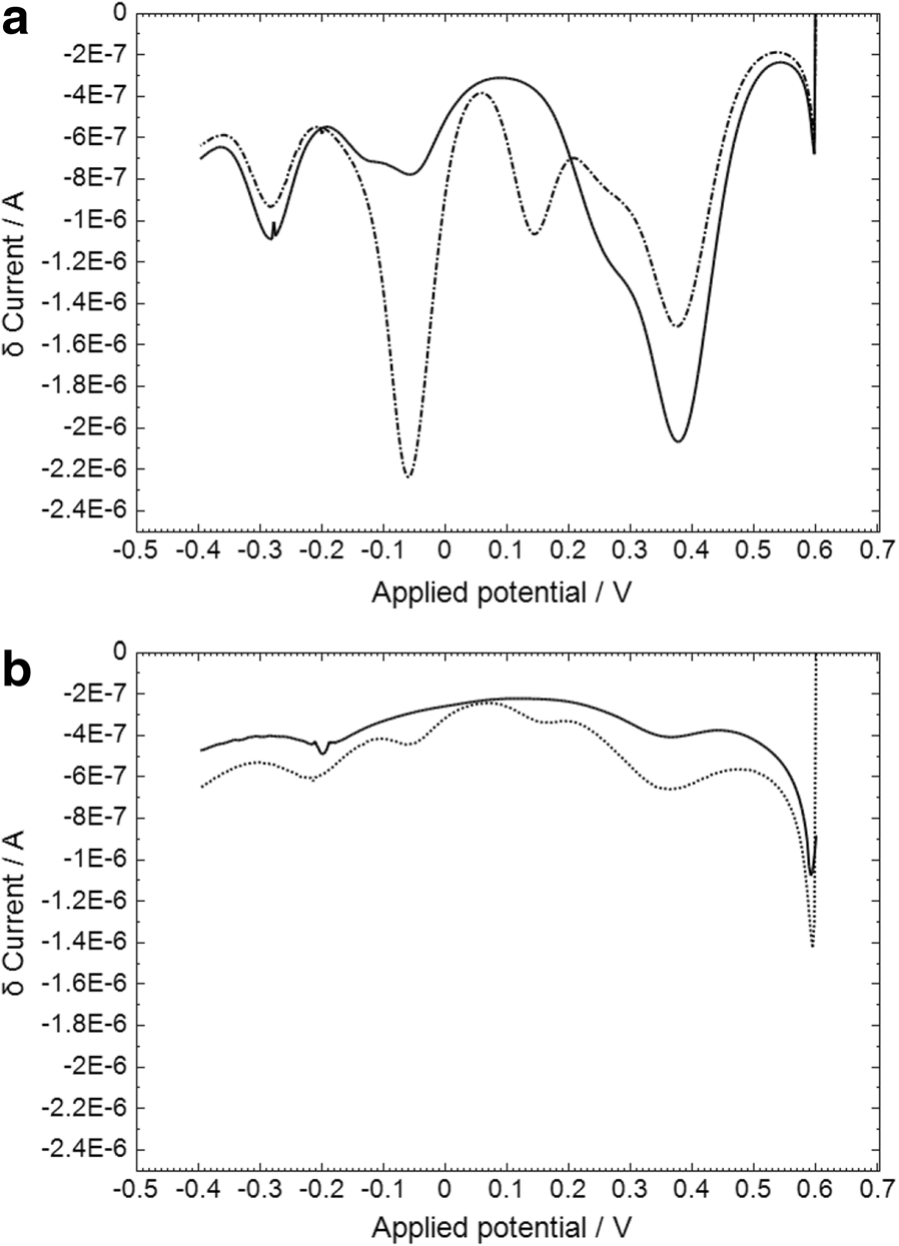
SWV response to MAMP in **a** saliva buffer or **b** saliva. The MAMP concentration was 0 μg/mL (*solid line*) or 5 μg/mL (*dashed line*). The overlayer was treated with 1.0 mg/mL of OX1006 in 0.4 M sodium carbonate buffer (pH 10.8), containing 1.0 M NaCl and 0.1 % TX-100. The SWV procedure consisted of a 10 s wait time after application of 7 μL of sample, then (1) galvanostatic oxidation at 800 nA for 30 s, (2) SWV with start voltage +0.6 V, stop voltage −0.4 V, 4.25 Hz frequency, 2.85 mV step potential and 50 mV amplitude

The square wave voltammetry (SWV) response to MAMP in saliva buffer or saliva using galvanostatic oxidation and the mediator overlayer is shown in Fig. 5. In saliva buffer, the main reduction peak at +0.38 V was reduced in the presence of MAMP (1800–1260 nA, 30 % reduction), and two new peaks were observed at +0.14 and −0.06 V (717 and 1430 nA). The reduction peak heights were very significantly reduced in saliva compared to saliva buffer, by approximately 85–95 % (peak heights at +0.34, +0.15 and −0.04 V were 205, 38 and 88 nA in the presence of 5 μg/mL MAMP). The overall response time with the SWV procedure was 122 s. Ideally the response time of the sensor would be in the range 15–30 s, although a response time of less than 120 s would still be acceptable for a roadside test as it would be considerably faster than the existing roadside tests. Therefore the electrochemical procedure was optimized for speed of response.

In order to increase the speed of the SWV technique, the first part of the scan between +0.6 and +0.1 V was conducted at a higher scan rate compared to the second part of the scan between +0.1 V and −0.4 V. Both parts of the scan were optimized for amplitude, step size and frequency. The third peak height is independent of frequency (Additional file 2), therefore a faster scan rate can be used for the first part of the scan without any adverse effect on the 3rd peak height.

**Fig. 6:**
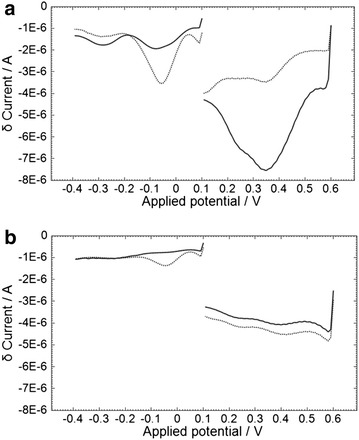
Split SWV response to MAMP in **a** saliva buffer or **b** saliva. The MAMP concentrations were 0 (*solid line*) or 5 μg/mL (*dashed line*). The overlayer was treated with 1.0 mg/mL of OX1006 in 0.4 M sodium carbonate buffer (pH 10.8), containing 1.0 M NaCl and 0.1 % TX-100. The SWV procedure consisted of a 10 s wait time after application of 7 μL of sample, then (1) galvanostatic oxidation at 800 nA for 30 s; (2) SWV-1 with start voltage +0.6 V, stop voltage +0.1 V, 20 Hz frequency, 10 mV step potential and 50 mV amplitude; (3) SWV-2 with start voltage +0.1 V, stop voltage −0.4 V, 4.25 Hz frequency, 10 mV step potential and 100 mV amplitude

**Fig. 7:**
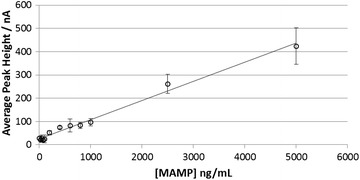
Calibration plot for response to MAMP in saliva obtained from a single donor, using the 3rd peak height obtained with the split SWV technique. Each sample was tested with 12 sensors. *Error bars* are 1 SD. The overlayer treatment and electrochemical procedure are described in Fig. 6

The split SWV responses to MAMP in saliva buffer and saliva are shown in Fig. 6, using frequencies of 20 and 4.25 Hz for the first and second parts of the scan. The new peak in response to MAMP is clearly observed at −0.06 V for saliva buffer and −0.04 V for saliva. The overall response time is 55 s. The calibration plot for response to MAMP in a saliva sample using the third peak of the optimized split SWV technique is shown in Fig. 7. Good linearity of response to MAMP was obtained (R^2^ 0.9877). The lower limit of detection was 400 ng/mL (0 ng/mL response +3 SD) which is considerably higher than that required for a commercial device (<10 ng/mL).

The LOD compares favourably with that obtained using indirect electrochemistry with 1,2-naphthoquinone-4-sulfonate [16], and it is considerably higher than the LODs obtained using direct electrochemical methods [13–15], although all these methods use aqueous solution and not undiluted saliva. Use of microelectrodes should provide greater sensitivity of response, since increased mass transport of MAMP to the electrode should result in increased peak heights i.e. higher nA per ng/mL MAMP. However this would require reproducible screen printed microelectrodes and development of a suitable manufacturing methodology was beyond the time and budgetary restraints of this work.

The response to MAMP and amphetamine in saliva using the split SWV technique showed a new peak formed in response to MAMP at −0.04 V, and no new peak observed in response to amphetamine (Additional file 3). This demonstrates the selectivity of the mediator to secondary amines over primary amines.

### Variation in response with different donor saliva samples

**Fig. 8:**
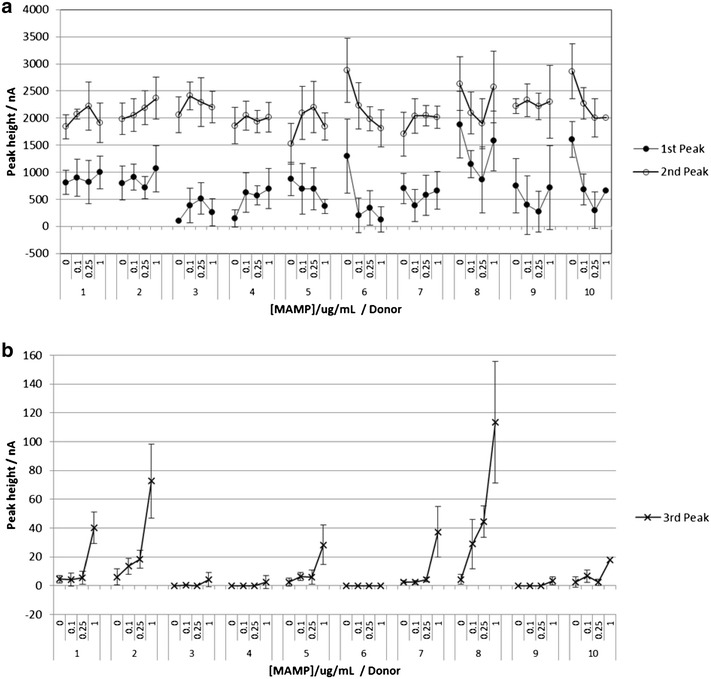
Donor variation in response to MAMP in saliva from 10 donors. **a** 1st and 2nd peak heights and **b** 3rd peak height. The MAMP concentrations were 0, 0.1, 0.25 and 1 μg/mL. Each sample was tested with 6 sensors. *Error bars* are 1 SD. The overlayer treatment and SWV procedure are described in Fig. 6

The response to saliva obtained from 10 donors is shown in Fig. 8. There is considerable variation in 1st and 3rd peak heights, and to a lesser extent the 2nd peak height, between the donors. At 0 μg/mL MAMP, the average peak heights range from 95 to 1878 nA for the 1st peak, 1523–2882 nA for the 2nd peak and 0–6 nA for the 3rd peak. At 1 μg/mL MAMP, the average peak heights range from 129 to 1578 nA for the 1st peak, 1813–2573 nA for the 2nd peak and 0–113 nA for the 3rd peak. The individual donor samples can give very different responses. For example, while the majority of the donor samples do not show a decrease in 1st and 2nd peak heights in response to MAMP, donors 6 and 10 do show a decrease in 1st and 2nd peak heights in response to MAMP (donor 6 gave 90 and 37 % decrease and donor 10 gave 59 and 30 % in 1st and 2nd peak heights, for response to 0 and 1 μg/mL MAMP). However for the 3rd peak, donor 6 gave no response to MAMP, whereas for donor 10 the 3rd peak height increased from 2.4 to 18 nA for 0–1 μg/mL MAMP. It can also be observed that only the samples from donors 2 and 8 show an increase in the 3rd peak height in response to 100 ng/mL MAMP (donor 2, 6.1–13.5 nA and donor 8, 4.3–28.9 nA for response to 0 and 100 ng/mL MAMP).

**Fig. 9:**
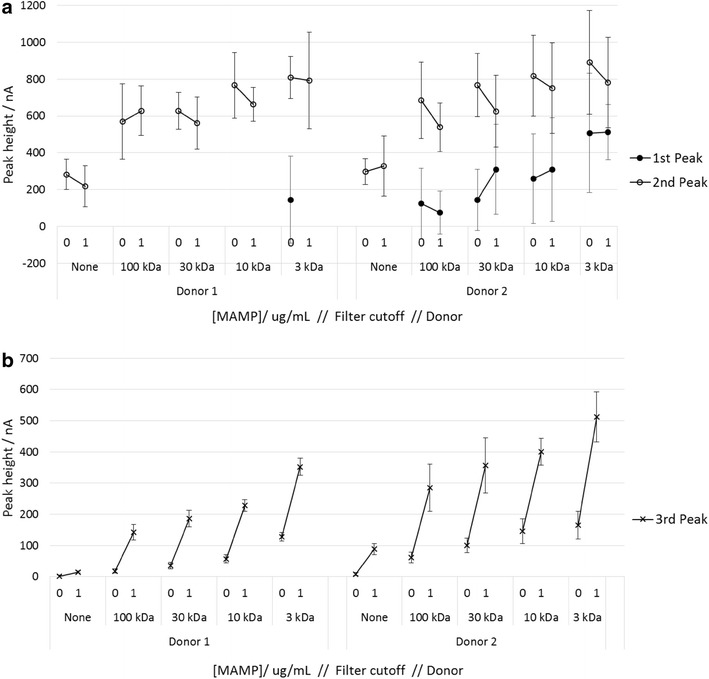
Response to MAMP in saliva from two donors, in unfiltered saliva and saliva filtrate. **a** 1st and 2nd peak heights and **b** 3rd peak height. Saliva filtrate was collected using centrifugal filters with 3, 10, 30 or 100 kDa cut-offs. The MAMP concentrations were 0 or 1 μg/mL. Each sample was tested with 6 sensors. *Error bars* are 1 SD. The overlayer treatment and SWV procedure is described in Fig. 6, except the galvanostatic current was 700 nA

To further investigate the effect of donor variation in saliva on response, saliva from two donors was centrifugally filtered using filters with cut-offs of 3, 10, 30 and 100 kDa. The results are shown in Fig. 9. There was a significant increase in the 2nd peak height and also in the 3rd MAMP peak height for 100 kDa filtered saliva compared to unfiltered saliva; with 1 μg/mL MAMP, the peak heights increase from 218 to 629 nA (donor 1, 2nd peak), and 15–142 nA (donor 1, 3rd peak), and from 329 to 539 nA (donor 2, 2nd peak) and 88–285 nA (donor 2, 3rd peak). This indicates high molecular weight species such as proteins and mucin have a significant negative impact on the peak height. For donor 1, the 1st peak is not present except for the 3 kDa filtered sample, while for donor 2 the 1st peak was not present for the unfiltered samples, but was present for the filtered samples.

The response to MAMP increased with decreasing molecular weight cut-off of the filter e.g. for donor 1, the 3rd peak heights in response to 1 μg/mL MAMP were 15, 142 and 353 nA for unfiltered saliva, 100 and 3 kDa filters. However there is still considerable donor variation in response with the filtrate from the 3 kDa filter (the 3rd peak heights for donors 1 and 2 were 353 and 512 nA respectively). While this filter will have removed larger proteins and mucins, some small proteins and protein fragments will remain, which may compete for adsorption sites on the electrode surface with the mediator MAMP adduct. In addition, the filtrate will contain endogenous amines which may react with the mediator.

The effect of the saliva components mucin and lysozyme on response are shown in Table 1. Addition of mucin had little effect, whereas addition of lysozyme resulted in significant reduction in peak heights, demonstrating the adverse effect of saliva proteins on response.

**Table 1:** Response to saliva buffer containing added protein

	Average peak height/nA (±1 SD)			% Decrease in peak height		
	1st peak (at +0.40 V)	2nd peak (at +0.25 V)	3rd peak (at −0.06 V)	1st peak	2nd peak	3rd peak
SSB	2865 ± 1369	3257 ± 1939	436 ± 30			
SSB + 0.021 mg/mL mucin	2665 ± 728	2581 ± 893	481 ± 59	−7.0	−20.8	10.4
SSB + 0.021 mg/mL mucin + 0.3 mg/mL lysozyme	1952 ± 1009	1781 ± 1018	382 ± 70	−26.7	−31.0	−20.5
SSB + 0.021 mg/mL mucin + 3.0 mg/mL lysozyme	985 ± 275	717 ± 233	54 ± 29	−49.5	−59.7	−85.8

(A) No addition and with the addition of (B) 0.021 mg/mL mucin; (C) 0.3 mg/mL lysozyme and 0.021 mg/mL mucin; and (D) 3 mg/mL lysozyme and 0.021 mg/mL mucin. The overlayer was treated with 0.2 mg/mL of OX1006 in 0.4 M sodium carbonate buffer (pH 10.8), containing 0.23 M NaCl and 0.1 % TX-100. Each sample was tested with 6 sensors. The SWV procedure is described in Fig. 6

## Experimental

(+)-Methamphetamine hydrochloride (M8750), d-amphetamine sulphate (A5880), human recombinant lysozyme (L1667) and mucin from bovine submaxillary glands (M3895) were obtained from Sigma-Aldrich Co. Ltd (Poole, UK). The mediators were synthesized by Peakdale Molecular (High Peak, UK). All other chemicals were purchased from Sigma-Aldrich Co. Ltd. All chemicals were used as received without further purification. All solutions were prepared using deionized water with resistivity no less than 18.2 MΩ cm.

Screen printed electrodes were fabricated in house with appropriate stencil designs using a DEK Horizon printing machine (DEK, Weymouth, UK). Successive layers of carbon-graphite ink (C2120403D1, modified in house by the addition of 0.1 % TX-100), dielectric ink (D2070423P5) and Ag/AgCl ink (60:40, C2030812P3) obtained from Gwent Electronic Materials Ltd. (Pontypool, UK) were printed onto a polyester substrate. The layers were cured using a tunnel drier at 70 °C (Natgraph, Nottingham, UK). The reproducibility of response of a sample of sensors from each print batch was determined using square wave voltammetry (SWV) with 1 mM OX1006 in 0.4 M sodium carbonate buffer (pH 10.8), 0.23 M NaCl, 0.0018 % TX-100. The SWV settings were as follows: start potential +0.6 V, stop potential −0.5 V, frequency 10 Hz, amplitude 0.05 V and step size 0.00285 V. Each sensor batch comprised 15 sheets with 4 rows of 48 sensors per sheet. A sample of 12 sensors from the second sheet of each batch were tested for SWV response to OX1006, and the responses were characterized for peak position and peak height. The %CVs were typically in the range 0.5–1.7 and 3–5 % for peak position and peak height respectively.

Voltammetric measurements were performed using either a MultiAutolab M101 or a μ-Autolab III potentiostat (Eco Chemie). The screen printed sensors were used as a two electrode system, with a combined counter/reference electrode (Ag/AgCl ink).

The overlayer material was composed of abaca and cellulosic fibres (75 %) in a polypropylene thermoplastic matrix (25 %), dry weight 16.5 g/m^2^ (CD020010, Ahlstrom) in reel format (1 cm wide) was obtained from Ahlstrom (Duns, UK). The overlayer was coated with OX1006 as follows: 1 mg/mL OX1006 was prepared in 0.4 M sodium carbonate buffer solution (pH 10.8) containing 1 M NaCl and 0.1 % Triton X-100. The solution was dispensed onto the membrane at a loading of 0.1–1 mg/mL and dried at 40 °C. The dried overlayer was heat soldered to each sensor along the edges.

Saliva buffer, which mimics real saliva except for the absence of proteins, consisted of 27.5 mM sodium chloride, 6.3 mM ammonium chloride, 4.9 mM sodium phosphate (monobasic), 2.9 mM potassium chloride, 1.1 mM sodium citrate (anhydrous), 0.02 mM magnesium chloride (anhydrous), 0.27 mM sodium carbonate and 0.2 mM calcium chloride.

Each saliva sample was collected immediately before use by spitting into a pot. Saliva samples containing MAMP were prepared by dissolving MAMP directly into the saliva sample at 1 mg/mL. Saliva samples containing lower MAMP concentrations were obtained by dilution of the 1 mg/mL sample with neat saliva.

Centrifugal filtration of saliva was performed using Amicon Ultra 0.5 mL centrifugal filters with molecular cut-off weights of 100, 30, 10, and 3 kDa. The samples were centrifuged at 14,000*g* for 10 min. The filters were weighed before and after centrifugation and deionised water was added to each filtrate to adjust for volume lost.

## Conclusions

The detection of 400 ng/mL MAMP in undiluted saliva has been reported using mediated disposable screen printed sensors with a response time of 55 s. While the response time is significantly faster than existing lateral flow immunodiagnostic tests, the limit of detection of the sensors is considerably higher (400 ng/mL compared to 10 ng/mL) and is too high to be acceptable as a screening test. The precision of the sensor response is adversely affected by saliva proteins and further development of the sensor is required to overcome these effects and obtain a commercially viable sensor. Saliva samples are notoriously variable in terms of composition and viscosity, even within the same donor sample collected over a short period of time, and it is probable that an on-strip dilution of the sample would decrease adverse effects arising from sample variability and viscosity, however this would require controlled sample dilution. It would also require greater sensitivity of response which may be achieved by the use of microelectrodes and this is a route that should be investigated further. In conclusion, development of a disposable roadside test for the rapid determination of methamphetamine in undiluted saliva is challenging, and requires significant further effort.

## Additional files

10.1186/s13065-016-0147-2 Mediator screen.
10.1186/s13065-016-0147-2 Effect of SWV-1 frequency.
10.1186/s13065-016-0147-2 Response to MAMP and AMP.

## Abbreviations

MAMP: (+)-methamphetamine
AMP: d-amphetamine
SWV: square wave voltammetry
DPV: differential pulse voltammetry
OX1006: *N*,*N*′-(2-nitro-1,4-phenylene)dibenzenesulfonamide
