# Supplementary material for: Disposable screen printed sensor for the electrochemical detection of methamphetamine in undiluted saliva
Source: Chem Cent J. 2016 Feb 1;10:3. doi: 10.1186/s13065-016-0147-2 (PMC4735951; doi:10.1186/s13065-016-0147-2)
Supplement: Supplementary file 1 — 10.1186/s13065-016-0147-2 Mediator screen. [file 13065_2016_147_MOESM1_ESM.docx]

**Additional file 1:**

**Mediator screen, incorporating Figures S1 and S2, and Table S1.**

An initial mediator screen was performed with several substituted N, N’-(1,4-phenylene)-dibenzenesulfonamide compounds, shown below in Figure S1. The responses of the various mediators to MAMP are summarized below in Table S1.

OX1001: No substituents

OX1002: b,b’ are -SO_2_Me

OX1003: d,d’ are –NO_2_

OX1004: c,c’ are –CO_2_H

OX1005: a is –OH

OX1006: a is -NO_2_

OX1007: a is NO_2_, b,b’ are –SO_2_Me

OX1009: a is –SO_3_H

OX1011: a is –CO_2_H

**Figure S1. Structures of the mediators**

**Table S1. Results of the mediator screen using DPV.** The experimental conditions for the mediator screen are described above.

| **Mediator** | **Parent reduction peak potential / V** | **Parent reduction peak height in response to 0 ug/mL MAMP / nA** | **Parent reduction peak height in response to 50 ug/mL MAMP / nA** | **% decrease in parent reduction peak height in response to 50 ug/mL MAMP** | **Peak potential of largest new peak in response to 50 ug/mL MAMP / V** | **Peak height of largest new peak in response to 0 ug/mL MAMP / nA** | **Peak height of largest new peak in response to 50 ug/mL MAMP / nA** | **Increase in new peak height / nA** |
| --- | --- | --- | --- | --- | --- | --- | --- | --- |
| **OX1001** | +0.22 | 4152 | 4104 | -1 | -0.16 | 0 | 1480 | +1480 |
| **OX1002** | +0.26 | 1625 | 1453 | -10 | -0.13 | 0 | 122 | +122 |
| **OX1003** | +0.27 | 2198 | 1823 | -17 | -0.16 | 123 | 469 | +346 |
| **OX1004** | +0.25 | 23 | 18 | -22 | -0.17 | 1 | 6 | +5 |
| **OX1005** | +0.11 | 2645 | 1938 | -27 | -0.15 | 8 | 30 | +24 |
| **OX1006** | +0.39 | 138 | 74 | -46 | -0.02 | 0 | 2258 | +1158 |
| **OX1007** | +0.44 | 36 | 29 | -19 | +0.02 | 0 | 129 | +129 |
| **OX1009** | +0.33 | 117 | 123 | +5 | +0.07 | 0 | 16 | +16 |
| **OX1011** | +0.30 | 131 | 100 | -24 | -0.06 | 2 | 15 | +13 |

The screen investigated two key parameters for selection of a mediator for the MAMP sensor: (1) formation of a large new peak in response to MAMP (>1000 nA); and (2) good potential separation between the peaks for the parent mediator and the new peak (>200 mV separation), to more easily enable peak height assignment. In addition, the mediator will preferably react with primary and secondary amines to give separate new peaks for each i.e. separate peaks at separate potentials which are well separated.

The DPV responses of several mediators showed more than one peak in the absence of MAMP, however in all cases there was one main peak. This was called the parent mediator peak and the parent mediator peak potentials and heights are given in Table S1. Similarly in the presence of MAMP, the DPV responses of almost all mediators showed more than one peak, comprising the parent mediator peak and in addition a new peak. In some instances, more than one new peak in response to MAMP was observed, and the peak height and position of the largest new peak is given in Table S1.

On this basis, the mediator screen led to the selection of two candidates, OX1001 and OX1006. It was found that OX1001 had a tendency to adsorb onto the electrode, most probably due to low solubility, which led to variable peak heights. Therefore OX1006 was selected.

The solubilities of OX1006 were determined to be 3.24, 33.27 and 249.11 ug/mL at pH 7.5, 8.5 and 9.5. At more alkaline conditions, OX1006 was determined in house to be soluble at 1 mg/mL in aqueous buffer (0.4M sodium carbonate (pH 10.8), 1M NaCl). The pKas of OX1006 were determined to be 6.05 and 8.00 (25°C). Therefore at pH 10.8, the mediator is fully deprotonated and highest solubility is achieved, and this pH was used for development of the sensor.

The DPV response of OX1006 to MAMP is shown in Figure S2. In the absence of MAMP, there was one main reduction peak at +0.40V. In the presence of MAMP, there is a reduction in the parent mediator peak height at +0.40V, and two new reduction peaks at +0.16V and -0.038V. The new reduction peak at -0.038V is considerably higher than the parent mediator peak height (552 and 1370 nA for 25 and 50 ug/mL MAMP compared to 205 nA for the parent mediator peak at +0.40V), and this is thought due to adsorption of the mediator-MAMP adduct onto the electrode surface.


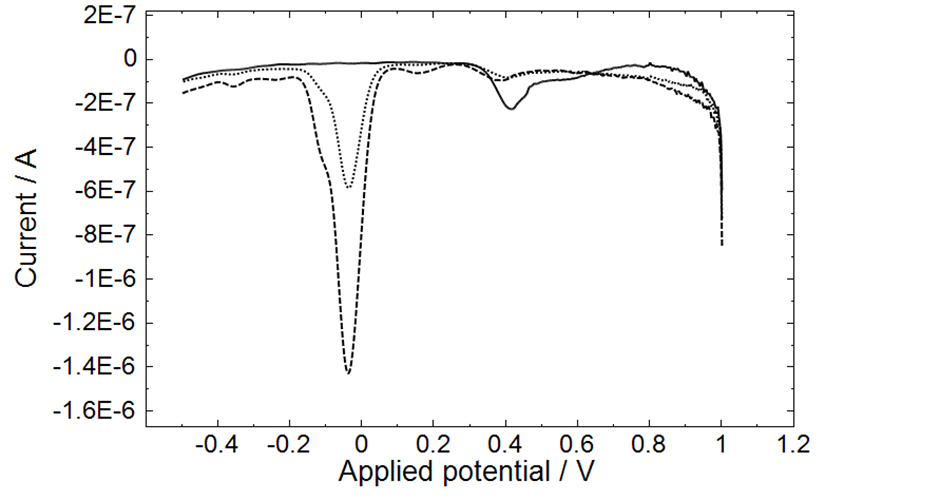


**Figure S2. DPV response of OX1006 mediator in the absence and presence of MAMP.** The MAMP concentration was 0, 25 or 50 ug/mL MAMP (solid line, dotted line and dashed line) in 0.1M sodium carbonate buffer (pH 10.4), 0.2M NaCl. 15uL of solution was pipetted onto the sensor. The DPV scan settings are given in the experimental section below.

**Experimental**

The mediator screen was performed as follows. Test solution was prepared by mixing 90 uL of solution A (0.2M AMPSO (pH 9.7), 0.2M NaCl) with 10 uL of solution B (1 mg/mL mediator in methanol) and 10 uL of solution A, C or D, where solutions C and D are 250 and 500 ug/mL methamphetamine in solution A. 15uL of test solution was pipetted onto a sensor and the DPV procedure started immediately. The DPV scan used a start potential of +1.0V, end potential of -0.5V, modulation amplitude of -0.05V, step potential of -0.00285V, interval time of 0.5 seconds and modulation time of 0.05 seconds.

The acid dissociation constants (pKa values) were determined by Sirius Analytical (Forest Row, UK) using a UV-metric method. The solubility of OX1006 was determined by Peakdale Molecular using the shake flask method using saturated solutions of mediator in 10% by volume of methanol solution to 90% by volume of 0.2M AMPSO (pH 7.5, 8.5 or 9.5), 0.2M NaCl. Analysis was by LC/MSMS

against a standard curve prepared in DMSO (in which the mediator was readily soluble).

Solubility data for OX1006 was obtained using the shake flask method by Peakdale Molecular. A saturated solution of OX1006 in 10% methanol: 90% aqueous 0.2M AMPSO, 0.2M NaCl solution, pH adjusted with NaOH to give pHs 7.5, 8.5 and 9.5 was used. Analysis was by LC/MSMS against a standard curve prepared in DMSO in which OX1006 was readily soluble.
