# Supplementary material for: Disposable screen printed sensor for the electrochemical detection of methamphetamine in undiluted saliva
Source: Chem Cent J. 2016 Feb 1;10:3. doi: 10.1186/s13065-016-0147-2 (PMC4735951; doi:10.1186/s13065-016-0147-2)
Supplement: Supplementary file 2 — 10.1186/s13065-016-0147-2 Effect of SWV-1 frequency. [file 13065_2016_147_MOESM2_ESM.docx]

**Additional file 2: Effect of SWV-1 frequency on 3^rd^ peak height**


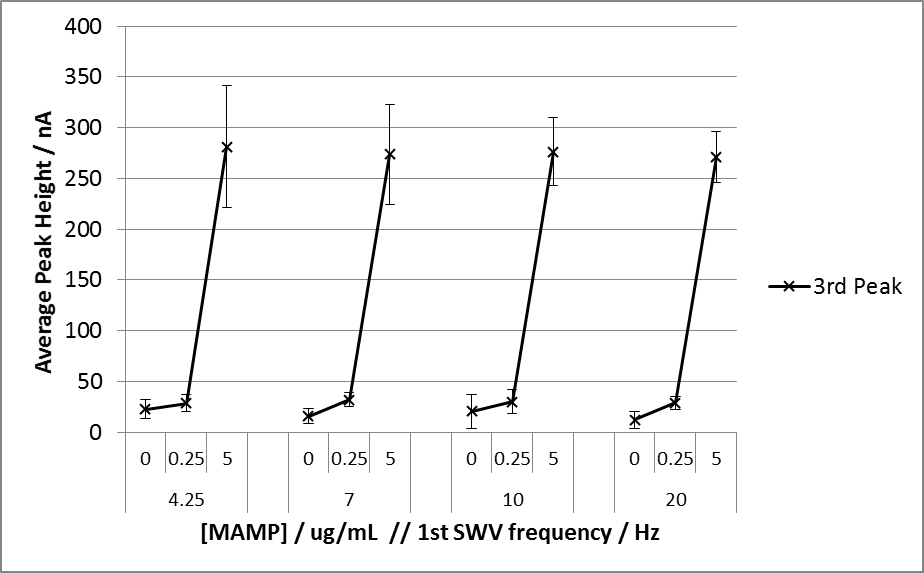


**Response to MAMP in saliva using the split SWV technique, varying the frequency of SWV-1.**

The 3^rd^ peak potential was -0.06V. Each sample was tested with 6 sensors. Error bars are one standard deviation. The overlayer was treated with 0.12 mg/mL of OX1006 in 0.4 M sodium carbonate buffer (pH 10.8), containing 0.23 M NaCl and 0.1% TX-100. The SWV procedure consisted of a 10 second wait time after application of 7 uL of sample, then (1) galvanostatic oxidation at 800 nA for 30 seconds; (2) SWV-1 with start voltage +0.6V, stop voltage +0.1V, 2.85 mV step potential, 50 mV amplitude and 4.25, 7, 10 or 20 Hz frequency; (3) SWV-2 with start voltage +0.1V, stop voltage -0.4V, 4.25 Hz frequency, 2.85 mV step potential and 50 mV amplitude.

**Figure S4. Split SWV response to MAMP and AMP in saliva.**


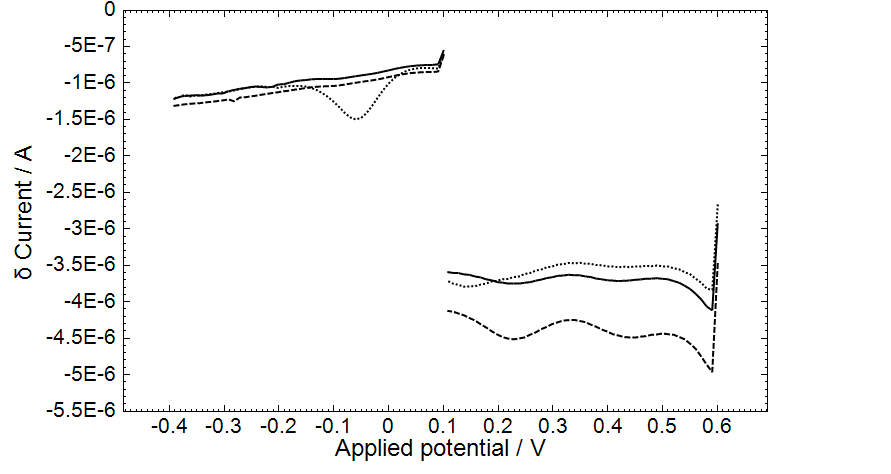


The substrate concentrations were 0 (solid line), 5 ug/mL MAMP (dotted line) or 5 ug/mL AMP (dashed line)**.** The overlayer was treated with 0.12 mg/mL of OX1006 in 0.4M sodium carbonate buffer (pH 10.8), containing 0.23M NaCl and 0.12% TX-100. The SWV procedure is described in Figure 9, except SWV-2 used 50 mV amplitude.
