# Supplementary material for: Disposable screen printed sensor for the electrochemical detection of methamphetamine in undiluted saliva
Source: Chem Cent J. 2016 Feb 1;10:3. doi: 10.1186/s13065-016-0147-2 (PMC4735951; doi:10.1186/s13065-016-0147-2)
Supplement: Supplementary file 3 — 10.1186/s13065-016-0147-2 Response to MAMP and AMP. [file 13065_2016_147_MOESM3_ESM.docx]

**Additional file 3: Split SWV response to MAMP and AMP in saliva**


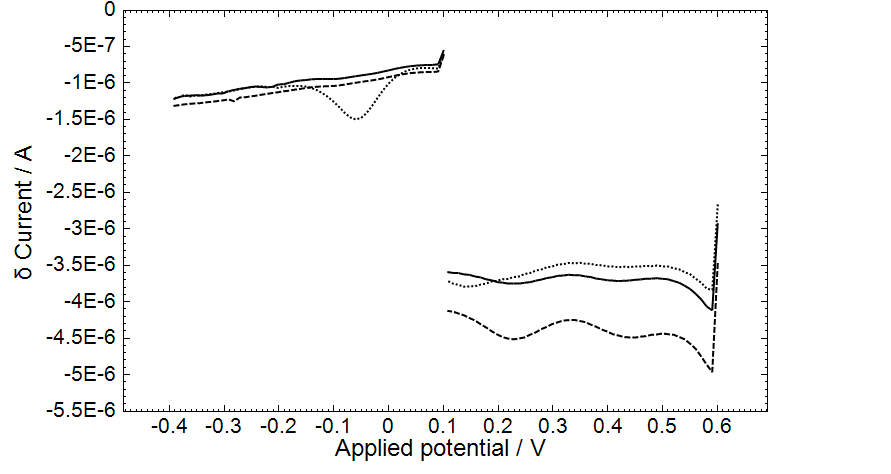


**Split SWV response to MAMP and AMP in saliva.** The substrate concentrations were 0 (solid line), 5 ug/mL MAMP (dotted line) or 5 ug/mL AMP (dashed line)**.** The overlayer was treated with 0.12 mg/mL of OX1006 in 0.4M sodium carbonate buffer (pH 10.8), containing 0.23M NaCl and 0.12% TX-100. The SWV procedure is described in Figure 6, except SWV-2 used 50 mV amplitude.
